# Insulin-like growth factor 1 predicts decompensation and long-term prognosis in patients with compensated cirrhosis

**DOI:** 10.3389/fmed.2023.1233928

**Published:** 2023-07-24

**Authors:** Chisato Saeki, Tomoya Kanai, Kaoru Ueda, Masanori Nakano, Tsunekazu Oikawa, Yuichi Torisu, Masayuki Saruta, Akihito Tsubota

**Affiliations:** ^1^Division of Gastroenterology and Hepatology, Department of Internal Medicine, The Jikei University School of Medicine, Tokyo, Japan; ^2^Division of Gastroenterology, Department of Internal Medicine, Fuji City General Hospital, Shizuoka, Japan; ^3^Project Research Units, Research Center for Medical Science, The Jikei University School of Medicine, Tokyo, Japan

**Keywords:** cirrhosis, insulin-like growth factor 1, liver functional reserve, prognosis, decompensation

## Abstract

**Aim:**

Insulin-like growth factor 1 (IGF-1), which is primarily produced in hepatocytes and is associated with liver functional reserve, plays a crucial role in the pathological condition of cirrhosis. This study aimed to investigate the usefulness of serum IGF-1 levels for predicting the long-term prognosis and decompensation development in patients with cirrhosis.

**Methods:**

We retrospectively evaluated 148 patients with cirrhosis and divided them into three groups according to baseline IGF-1 levels: low (L)-, intermediate (I)-, and high (H)-IGF-1 groups. The cumulative survival rates were compared among these groups in compensated and decompensated cirrhosis, respectively. Significant and independent factors associated with mortality and decompensation development were identified using Cox proportional hazards regression analysis.

**Results:**

The median observation period was 57.1 (41.7–63.2) months. Thirty (20.3%) patients died of liver disease-related events and 21 (22.3%) patients with compensated cirrhosis developed decompensation. Multivariate analysis identified low serum IGF-1 levels as a significant and independent factor associated with mortality (all patients: hazard ratio [HR], 0.967; *p* = 0.004; patients with compensated cirrhosis: HR, 0.927; *p* = 0.002). The cumulative survival rates were significantly lower in the L-IGF-1 group than in the H-IGF-1 and I-IGF-1 groups (all patients: *p* < 0.001 and = 0.009; patients with compensated cirrhosis: *p* = 0.012 and 0.003, respectively). However, in decompensated cirrhosis, the cumulative survival rates demonstrated no significant differences among the three groups. The cumulative decompensation incidence rates were significantly higher in the L-IGF-1 group than in the H-IGF-1 and I-IGF-1 groups (*p* < 0.001 and = 0.009, respectively). Low serum IGF-1 levels were significantly and independently associated with decompensation development (HR, 0.939; *p* < 0.001).

**Conclusion:**

Low serum IGF-1 levels were significantly and independently associated with decompensation development and poor long-term prognosis in patients with compensated cirrhosis. Therefore, IGF-1 may be useful for predicting decompensation-related events and should be regularly monitored in the management of compensated phase.

## 1. Introduction

Cirrhosis is the end stage of chronic liver disease (CLD) with different etiology, and it is a major cause of morbidity and mortality, leading to over 1.32 million (2.4%) global deaths (1, 2). It progresses from a compensated phase (in which most cases are asymptomatic) to a decompensated phase (in which variceal hemorrhage, ascites, encephalopathy, and jaundice can occur) (3). A better prognosis was observed in patients with compensated than those with decompensated cirrhosis, with median survival times of >12 years and < 2 years, respectively (3). Therefore, the management of patients with compensated cirrhosis is crucial to inhibit decompensated cirrhosis progression and thereby reduce the mortality rate. The Child–Pugh (CP) classification, as determined based on serum albumin, total bilirubin, and prothrombin time (PT) levels, ascites degree, and encephalopathy grade, is widely used to evaluate liver functional reserve (4). This classification can reflect clinical features and estimate patients’ prognoses. A better prognosis was observed in patients with CP class A (nearly corresponding to compensated cirrhosis) than those with CP class B/C (nearly corresponding to decompensated cirrhosis) (3). However, this scoring system includes subjective components, i.e., ascites and encephalopathy, and therefore may reduce clinical assessment reliability and survival prediction accuracy (5). Furthermore, it can hardly assess prognosis in patients with compensated cirrhosis (6).

Insulin-like growth factor 1 (IGF-1), which is primarily produced in hepatocytes and involved in mediating growth and metabolism, plays a crucial role in the pathological condition of CLD (7–9). Its circulating levels have been reported to correlate with liver functional reserve. They are decreased with advanced disease stage and malnutrition status, thereby exacerbating or causing insulin resistance, reactive oxygen species, inflammation, mitochondrial dysfunction, liver fibrosis, osteoporosis, and sarcopenia (7–12). A previous study revealed significantly lower 2 years survival rates in inpatients with cirrhosis with low plasma IGF-1 levels than those with high plasma IGF-1 levels (13). Furthermore, combined serum IGF-1 levels and CP scores (IGF-CP score) more accurately predict 1 year mortality than CP or model for end-stage liver disease (MELD) scores alone in patients with decompensated cirrhosis (5). Thus, circulating IGF-1 levels may be useful for estimating liver functional reserve or pathological condition and predicting prognosis in patients with cirrhosis. However, previous studies were limited to decompensated cirrhosis or short-term research duration and have not yet reported the association of IGF-1 with long-term prognosis in patients with compensated and decompensated cirrhosis, respectively.

This study aimed to investigate the usefulness of serum IGF-1 levels for predicting the long-term prognosis of patients with cirrhosis and the difference in the predictive performance of IGF-1 between patients with compensated and decompensated cirrhosis. Furthermore, we evaluated the usefulness of serum IGF-1 levels for predicting decompensation development in patients with compensated cirrhosis.

## 2. Materials and methods

### 2.1. Study participants

This retrospective study included 148 consecutive patients with cirrhosis who presented to the Jikei University School of Medicine (Tokyo, Japan) and Fuji City General Hospital (Shizuoka, Japan) between 2017 and 2020. Inclusion criteria were (i) diagnosis of cirrhosis caused by any etiology and (ii) available baseline serum IGF-1 measurements. Exclusion criteria were (i) preexisting malignancies, including hepatocellular carcinoma (HCC); (ii) liver transplantation history; and (iii) acute liver failure. Cirrhosis diagnosis was based on laboratory tests and imaging/endoscopic findings, such as surface nodularity, liver deformity with right lobe shrinkage and left lobe enlargement, ascites, splenomegaly, portosystemic collateral, and esophageal/gastric varices (14). Decompensated cirrhosis was diagnosed by the development of variceal hemorrhage, ascites, encephalopathy, and jaundice (15). Liver functional reserve was evaluated using the MELD score and CP classification (4, 16). The endpoint of this study was death from liver disease-related events. Patients who underwent liver transplantation for liver failure were considered as death and those who died from non-liver-related causes were considered censored cases. This study complied with the 2013 Declaration of Helsinki and was approved by the ethics committees of the Jikei University School of Medicine (approval number: 34-021) and Fuji City General Hospital (approval number: 279).

### 2.2. Laboratory assessments

Serum albumin, total bilirubin, creatinine, sodium, Mac-2 binding protein glycosylation isomer (M2BPGi, a hepatic fibrosis marker), and PT were measured using standard laboratory methods. Serum IGF-1 levels were evaluated using an immunoradiometric assay (Fujirebio, Tokyo, Japan).

### 2.3. Patient classification based on baseline serum IGF-1 levels

The median baseline IGF-1 levels of all patients and those with compensated and decompensated cirrhosis were 54 (interquartile range, 41–74), 61 (47–78), and 46 (32–61) ng/mL, respectively (Supplementary Figure S1). Patients were classified into three groups according to the quartiles: low (L)-IGF1_all_ group (≤41 ng/mL), intermediate (I)-IGF1_all_ group (41–74 ng/mL), and high (H)-IGF1_all_ group (≥74 ng/mL) for all patients; L-IGF1_com_ group (≤47 ng/mL), I-IGF1_com_ group (47–78 ng/mL), and H-IGF1_com_ group (≥78 ng/mL) for those with compensated cirrhosis; and L-IGF1_deco_ group (≤32 ng/mL), I-IGF1_deco_ group (32–61 ng/mL), and H-IGF1_deco_ group (≥61 ng/mL) for those with decompensated cirrhosis.

### 2.4. Statistical analysis

Between-group differences were compared using the Mann–Whitney *U* test or the Kruskal–Wallis test followed by the Steel–Dwass *post hoc* test, as appropriate, for continuous variables that were presented as medians (interquartile ranges), and the chi-squared test for categorical variables that were presented as numbers (percentages). Correlations between IGF-1 and continuous variables were analyzed using Spearman’s rank correlation test. The cumulative survival rates were estimated using the Kaplan–Meier method, and the between-group differences were compared using the log-rank test and Bonferroni multiple-comparison method. Significant and independent factors associated with mortality were identified using univariate and multivariate Cox proportional hazards models. SPSS Statistics version 27 (IBM Japan, Tokyo, Japan) was used for all statistical analyses. Values of *p* < 0.05 were considered statistically significant.

## 3. Results

### 3.1. Patient characteristics

Table 1 summarizes the baseline clinical characteristics of 148 included patients. The median age was 69 (57.0–76.0) years, and 96 (64.9%) patients were men. The median MELD score was 8.0 (7.0–11.0). The prevalence of decompensated cirrhosis was 36.5% (54/148). Serum IGF-1 levels in patients with decompensated cirrhosis were significantly lower than in those with compensated cirrhosis (median, 46 ng/mL vs. 61 ng/mL; *p* < 0.001; Supplementary Figure S1). Additionally, they significantly differed among CP classes (*p* < 0.001; Supplementary Figure S2) and were significantly higher in patients with the CP class A (median, 62 ng/mL) than in those with the CP class B (median, 43 ng/mL; *p* < 0.001) and class C (median, 34 ng/mL; *p* = 0.005) in the *post hoc* analysis. However, when the patients were divided into three groups according to age (<65, 65–74, and ≥75 years), serum IGF-1 levels did not significantly differ among the three groups (*p* = 0.738; Supplementary Figure S3).

**Table 1:** Characteristics of the three groups based on serum IGF-1 levels.

Variable	ALL	L-IGF1_all_	I-IGF1_all_	H-IGF1_all_	*p-*value
Patients, *n* (%)	148	41 (27.7)	70 (47.3)	37 (25.0)	
Man, *n* (%)	96 (64.9)	27 (65.9)	43 (61.4)	26 (70.3)	0.652
Age (years)	69.0 (57.0–76.0)	66.0 (53.5–76.0)	73.0 (59.0–76.5)	64.0 (55.0–73.5)	0.085
BMI (kg/m^2^)	23.6 (21.5–26.1)	22.5 (20.4–25.7)	23.6 (21.3–26.0)	24.8 (22.4–26.8)	0.083
*Etiology*					
HBV/HCV/alcohol/other, *n*	14/38/58/38	2/12/21/6	6/17/21/26	6/9/16/6	0.042
Decompensated cirrhosis, *n* (%)	54 (36.5)	23 (56.1)	25 (35.7)	6 (16.2)	0.001
Child–Pugh score	6 (5–7)	7 (5–8)	6 (5–7)	5 (5–6)	<0.001
MELD score	8.0 (7.0–11.0)	10.0 (8.0–13.0)	8.0 (7.0–10.0)	7.0 (6.0–10.0)	<0.001
Total bilirubin (mg/dL)	0.8 (0.6–1.3)	1.0 (0.7–2.0)	0.8 (0.5–1.2)	0.7 (0.6–1.1)	0.014
Albumin (g/dL)	3.8 (3.3–4.2)	3.4 (3.2–3.9)	3.9 (3.3–4.1)	4.2 (3.7–4.4)	<0.001
Creatinine (mg/dL)	0.9 (0.7–1.1)	0.8 (0.7–1.1)	0.9 (0.7–1.1)	0.9 (0.8–1.2)	0.148
Sodium (mEq/L)	140 (138–141)	139 (137–140)	140 (138–142)	140 (139–142)	0.061
Prothrombin time (%)	81 (68–100)	63 (50–81)	83 (71–99)	91 (79–100)	<0.001
IGF-1 (ng/mL)	54 (41–74)	32 (26–39)	55 (49–63)	87 (78–102)	<0.001
M2BPGi (C.O.I.)	2.79 (1.36–5.56)	4.47 (2.88–8.21)	2.66 (1.63–4.74)	1.19 (0.86–3.25)	<0.001

Continuous variables are shown as median (interquartile range). Statistical analysis was performed using the chi-squared test or the Kruskal–Wallis test, as appropriate. BMI, body mass index; C.O.I., cut-off index; HBV, hepatitis B virus; HCV, hepatitis C virus; IGF-1, insulin-like growth factor 1; M2BPGi, Mac-2 binding protein glycosylation isomer; MELD, model for end-stage liver disease; L-IGF1_all_, low-IGF-1 group; I-IGF1_all_, intermediate-IGF-1 group; H-IGF1_all_, high-IGF-1 group.

### 3.2. Clinical characteristics of patients according to baseline serum IGF-1 levels

The L-IGF1_all_, I-IGF1_all_, and H-IGF1_all_ group distributions were 27.7% (41/148), 47.3% (70/148), and 25.0% (37/148), respectively (Table 1). Etiology, decompensated cirrhosis prevalence, M2BPGi, and liver functional reserve-related factors (CP score, MELD score, total bilirubin, albumin, and PT) significantly differed among the three groups.

### 3.3. Correlations between IGF-1 and liver functional reserve-related factors and fibrosis marker

**Figure 1:**
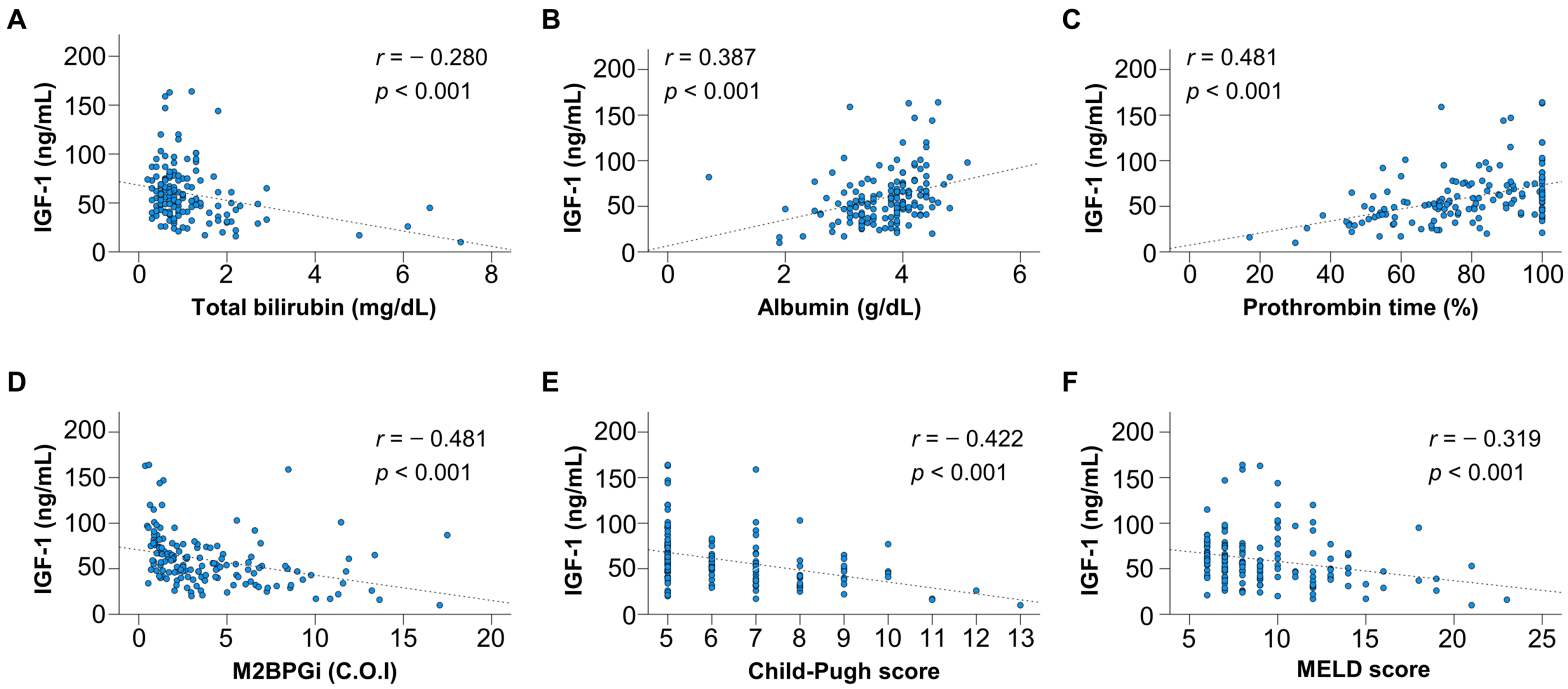
Correlation of insulin-like growth factor 1 (IGF-1) with liver functional reserve-related parameters and fibrosis marker. IGF-1 significantly correlated with **(A)** total bilirubin, **(B)** albumin, **(C)** prothrombin time, **(D)** Mac-2 binding protein glycosylation isomer (M2BPGi), **(E)** Child–Pugh score, and **(F)** model for end-stage liver disease (MELD) score (*p* < 0.001 for all).

The correlations between IGF-1 and liver functional reserve-related factors or M2BPGi were investigated using Spearman’s rank correlation test (Figures 1A–F). Serum IGF-1 levels significantly correlated with total bilirubin (*r* = −0.280), albumin (*r* = 0.387), PT (*r* = 0.481), and M2BPGi (*r* = −0.481) levels, CP score (*r* = −0.422), and MELD score (*r* = −0.319) (*p* < 0.001 for all).

### 3.4. Comparison of cumulative survival rates according to baseline serum IGF-1 levels

**Figure 2:**
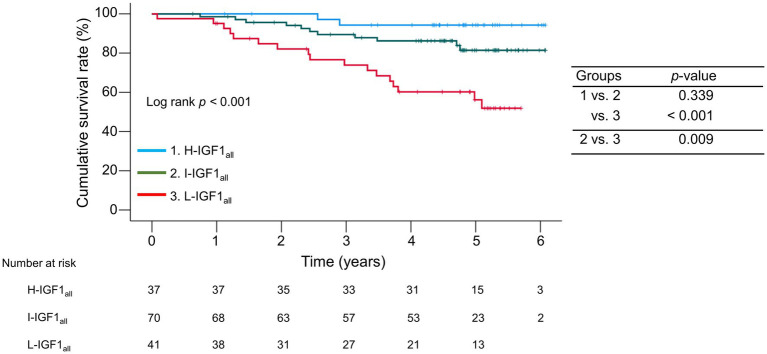
Comparison of the cumulative survival rates among the three groups stratified following the baseline serum levels of insulin-like growth factor 1 (IGF-1) levels. The cumulative survival rates were significantly lower in the low-IGF-1 (L-IGF1_all_) group than in the high-IGF-1(H-IGF1_all_) and intermediate-IGF-1(I-IGF1_all_) groups (*p* < 0.001 and = 0.009, respectively).

The median observation period was 57.1 (41.7–63.2) months. During the follow-up period, 30 (20.3%) patients died of liver disease-related events, including liver failure (*n* = 21), rupture of esophageal varices (*n* = 6), liver transplantation (*n* = 2), and hepatocellular carcinoma (*n* = 1). The 1-, 3-, and 5 years cumulative survival rates were 95.1, 73.9, and 56.2% in the L-IGH1_all_ group; 98.6, 89.5, and 81.4% in the I-IGH1_all_ group; and 100.0, 94.3, and 94.3% in the H-IGH1_all_ group (Figure 2). The cumulative survival rates were significantly lower in the L-IGF1_all_ group than in the H-IGF1_all_ and I-IGF1_all_ groups (*p* < 0.001 and = 0.009, respectively). The L-IGF1_all_ group had significantly lower cumulative survival rates than the H-IGF1_all_ and I-IGF1_all_ groups when nine patients who died from non-liver-related causes (L-IGF1_all_, *n* = 6; I-IGF1_all_, *n* = 2; H-IGF1_all_, *n* = 1) were considered the same as liver-related deaths (*p* < 0.001 for both; Supplementary Figure S4).

**Figure 3:**
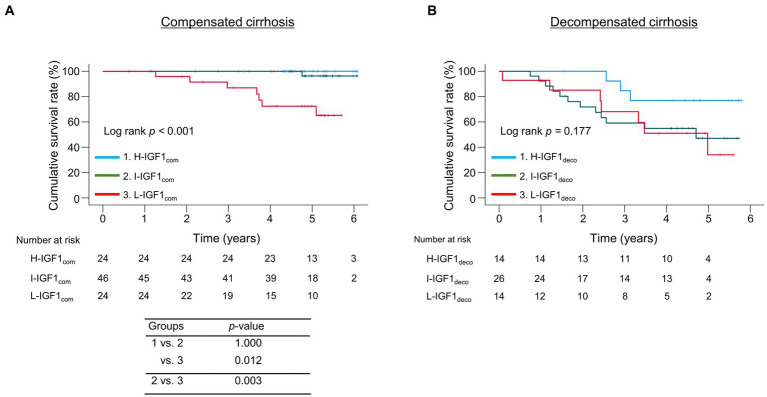
Comparison of the cumulative survival rates among the three groups stratified following the baseline serum levels of insulin-like growth factor 1 (IGF-1) levels in compensated or decompensated cirrhosis. **(A)** In compensated cirrhosis, the cumulative survival rates were significantly lower in the low-IGF-1 (L-IGF1_com_) group than in the high-IGF-1 (H-IGF1_com_) and intermediate-IGF-1 (I-IGF1_com_) groups (*p* = 0.012 and 0.003, respectively). **(B)** In decompensated cirrhosis, the cumulative survival rates did not significantly differ among the three groups (*p* = 0.177).

We divided the patients into the compensated and decompensated cirrhosis groups and compared the cumulative survival rates among the three IGF1 groups in each cirrhosis group (Figure 3). The 1-, 3-, and 5 years cumulative survival rates in the compensated cirrhosis group were 100.0, 86.9, and 72.4% in the L-IGF1_com_ group; 100.0, 100, and 96.3% in the I-IGF1_com_ group; and 100.0, 100.0, and 100.0% in the H-IGF1_com_ group (Figure 3A). The L-IGF1_com_ group had significantly lower cumulative survival rates than the H-IGF1_com_ and I-IGF1_com_ groups (*p* = 0.012 and 0.003, respectively). Meanwhile, the 1-, 3-, and 5 years cumulative survival rates in the decompensated cirrhosis group were 92.9, 68.1, and 34.0% in the L-IGF1_deco_ group; 92.3, 59.1, and 47.1% in the I-IGF1_deco_ group; and 100.0, 84.6, and 76.9% in the H-IGF1_deco_ group (Figure 3B). These rates demonstrated no significant differences among the three groups (*p* = 0.177). However, the redivision from 3 to 2 groups (H-IGF1_deco_ versus combined I-IGF1_deco_ and L-IGF1_deco_) demonstrated a marginally significant difference in the cumulative survival rates between the two groups (*p* = 0.064; Supplementary Figure S5).

### 3.5. Prognostic factors in patients with cirrhosis

Univariate analysis revealed that the following variables were significantly associated with mortality: decompensated cirrhosis, CP score, MELD score, total bilirubin, albumin, sodium, PT, IGF-1, and M2BPGi in all patients (Supplementary Table S1); PT, IGF-1, and M2BPGi in those with compensated cirrhosis (Supplementary Table S2); and CP score, MELD score, total bilirubin, albumin, IGF-1, and M2BPGi in those with decompensated cirrhosis (Supplementary Table S3). Cox proportional hazards regression analysis identified the following variables as significant and independent prognostic factors: high CP score [hazard ratio (HR), 1.248; 95% confidence interval (CI), 1.014–1.538; *p* = 0.037], low albumin levels (HR, 0.346; 95% CI, 0.204–0.589; *p* < 0.001), and low IGF-1 levels (HR, 0.967; 95% CI, 0.945–0.989; *p* = 0.004) in all patients (Table 2); low IGF-1 levels (HR, 0.927; 95% CI, 0.884–0.972; *p* = 0.002) in those with compensated cirrhosis (Table 3); and low albumin levels (HR, 0.097; 95% CI, 0.038–0.245; *p* < 0.001) in those with decompensated cirrhosis (Table 4).

**Table 2:** Significant factors associated with mortality in all patients.

Variable	Univariate		Multivariate	
	HR (95%CI)	*p-*value	HR (95%CI)	*p-*value
Decompensated cirrhosis	6.664 (2.955–15.029)	<0.001		
Child–Pugh score	1.695 (1.454–1.977)	<0.001	1.248 (1.014–1.538)	0.037
MELD score	1.202 (1.106–1.305)	<0.001		
Total bilirubin (mg/dL)	1.606 (1.296–1.991)	<0.001		
Albumin (g/dL)	0.376 (0.274–0.515)	<0.001	0.346 (0.204–0.589)	< 0.001
Sodium (mEq/L)	0.796 (0.688–0.920)	0.002		
Prothrombin time (%)	0.969 (0.951–0.987)	<0.001		
IGF-1 (ng/mL)	0.952 (0.932–0.973)	<0.001	0.967 (0.945–0.989)	0.004
M2BPGi (C.O.I.)	1.208 (1.125–1.297)	<0.001		

CI, confidence interval; C.O.I., cut-off index; HR, hazard ratio; IGF-1, insulin-like growth factor 1; M2BPGi, Mac-2 binding protein glycosylation isomer; MELD, model for end-stage liver disease.

**Table 3:** Significant factors associated with mortality in patients with compensated cirrhosis.

Variable	Univariate		Multivariate	
	HR (95%CI)	*p-*value	HR (95%CI)	*p-*value
Prothrombin time (%)	0.957 (0.915–1.001)	0.054		
IGF-1 (ng/mL)	0.927 (0.884–0.972)	0.002	0.927 (0.884–0.972)	0.002
M2BPGi (C.O.I.)	1.418 (1.008–1.996)	0.045		

CI, confidence interval; C.O.I., cut-off index; HR, hazard ratio; IGF-1, insulin-like growth factor 1; M2BPGi, Mac-2 binding protein glycosylation isomer.

**Table 4:** Significant factors associated with mortality in patients with decompensated cirrhosis.

Variable	Univariate		Multivariate	
	HR (95%CI)	*p-*value	HR (95%CI)	*p-*value
Child–Pugh score	1.548 (1.218–1.968)	<0.001		
MELD score	1.113 (1.005–1.233)	0.039		
Total bilirubin (mg/dL)	1.286 (1.002–1.651)	0.048		
Albumin (g/dL)	0.104 (0.041–0.265)	<0.001	0.097 (0.038–0.245)	<0.001
IGF-1 (ng/mL)	0.974 (0.951–0.998)	0.034		
M2BPGi (C.O.I.)	1.086 (0.985–1.196)	0.097		

CI, confidence interval; C.O.I., cut-off index; HR, hazard ratio; IGF-1, insulin-like growth factor 1; M2BPGi, Mac-2 binding protein glycosylation isomer; MELD, model for end-stage liver disease.

### 3.6. Comparison of cumulative incidence of decompensation according to baseline serum IGF-1 levels

**Figure 4:**
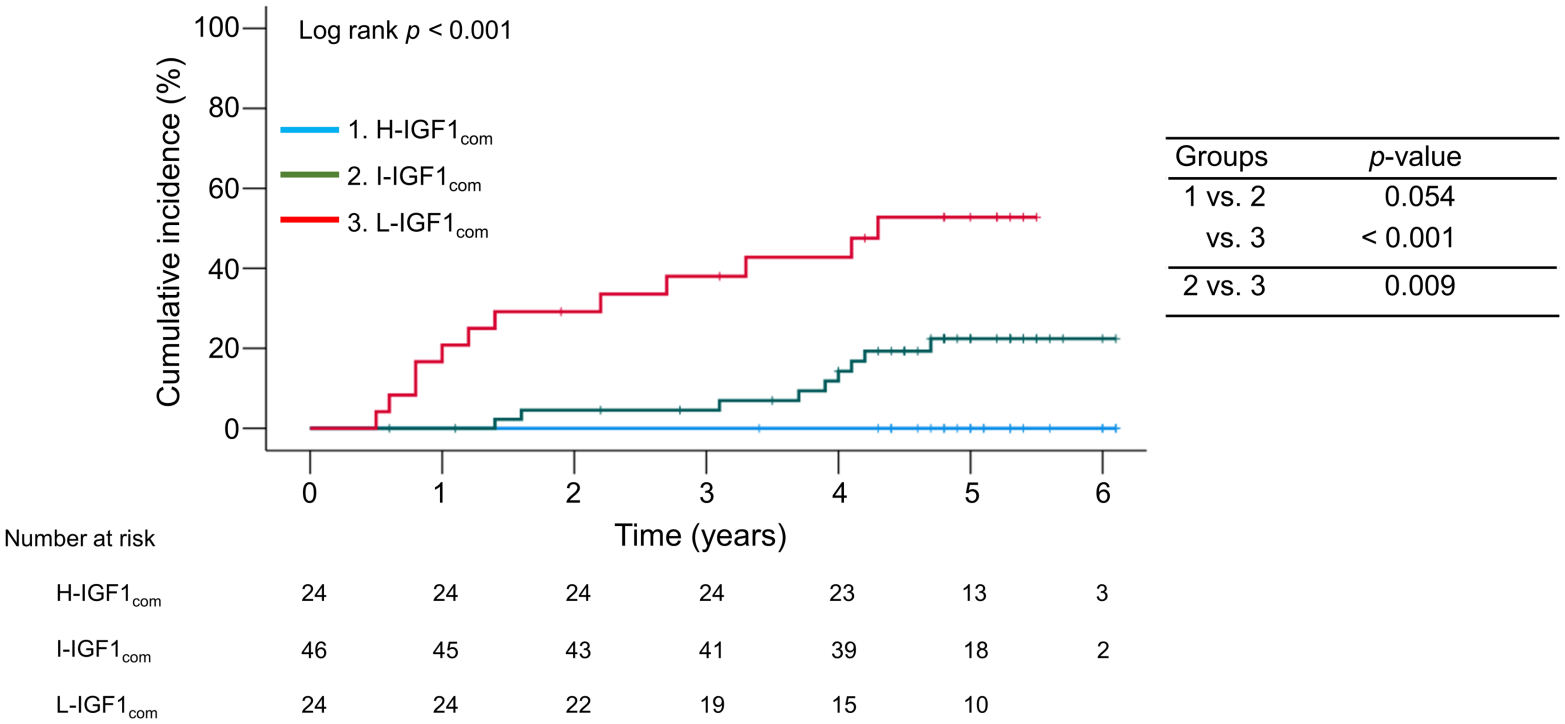
Comparison of the cumulative incidence rates of decompensation among the three groups stratified following the baseline serum insulin-like growth factor 1 (IGF-1) levels in compensated cirrhosis. The cumulative incidence rates of decompensation were significantly higher in the low-IGF-1 (L-IGF1_com_) group than in the high-IGF-1 (H-IGF1_com_) and intermediate-IGF-1 (I-IGF1_com_) groups (*p* < 0.001 and = 0.009, respectively).

During the follow-up period, 21 (22.3%) patients with compensated cirrhosis developed decompensation. The 1-, 3-, and 5 years cumulative incidence rates of decompensation were 20.8, 38.0, and 52.8% in the L-IGH1_com_ group; 0.0, 4.5, and 22.4% in the I-IGH1_com_ group; and 0.0, 0.0, and 0.0% in the H-IGH1_com_ group (Figure 4). The cumulative decompensation incidence rates were significantly higher in the L-IGH1_com_ group than in the H-IGH1_com_ and I-IGH1_com_ groups (*p* < 0.001 and = 0.009, respectively).

### 3.7. Factors associated with decompensation development

In the univariate analysis, CP score, total bilirubin, albumin, PT, IGF-1, and M2BPGi were significantly associated with decompensation development in patients with compensated cirrhosis (Supplementary Table S4). Cox proportional hazards regression analysis identified low albumin levels (HR, 0.447; 95% CI, 0.223–0.896; *p* = 0.023) and low IGF-1 levels (HR, 0.939; 95% CI, 0.913–0.966; *p* < 0.001) as significant and independent factors associated with decompensation development (Table 5).

**Table 5:** Significant factors associated with development of decompensation.

Variable	Univariate		Multivariate	
	HR (95%CI)	*p-*value	HR (95%CI)	*p-*value
Child–Pugh score	2.932 (1.623–5.295)	<0.001		
Total bilirubin (mg/dL)	3.065 (1.178–7.975)	0.022		
Albumin (g/dL)	0.603 (0.373–0.974)	0.039	0.447 (0.223–0.896)	0.023
Prothrombin time (%)	0.958 (0.932–0.985)	0.003		
IGF-1 (ng/mL)	0.942 (0.917–0.968)	<0.001	0.939 (0.913–0.966)	<0.001
M2BPGi (C.O.I.)	1.348 (1.097–1.655)	0.004		

CI, confidence interval; C.O.I., cut-off index; HR, hazard ratio; IGF-1, insulin-like growth factor 1; M2BPGi, Mac-2 binding protein glycosylation isomer.

## 4. Discussion

Most patients with compensated cirrhosis are asymptomatic and have a better prognosis compared to those with decompensated cirrhosis (3). However, various liver disease-related events develop and mortality increases evidently during liver disease progression from the compensated phase to the decompensated phase (15). Therefore, early decompensation prediction and medical countermeasures against complications are crucial in patients with compensated cirrhosis. IGF-1 can inhibit or improve hepatic inflammation and fibrosis, and therefore, may be deeply involved in the pathological CLD condition (7–9). Reportedly, circulating IGF-1 levels decrease with disease progression, and the decreased levels are associated with poor short-term prognosis in patients with cirrhosis, especially decompensated cirrhosis (5, 13). The present study is the first to report the association between serum IGF-1 levels and long-term prognosis in patients with compensated and decompensated cirrhosis, respectively. Intriguingly, serum IGF-1 levels were the only significant prognostic factor in patients with compensated cirrhosis, whereas serum albumin (but not IGF-1) levels in those with decompensated cirrhosis. Notably, deaths were not observed, as well as cases with decompensation development in the H-IGF1_com_ group when limited to patients with compensated cirrhosis. Meanwhile, the H-IGF1_deco_ group in patients with decompensated cirrhosis also had the highest survival rates, although marginally significant, and all H-IGF1_deco_ patients survived during the first 2 years.

One study of patients with cirrhosis (without HCC: *n* = 64; CP class B/C, 73.4%) revealed that plasma IGF-1 levels (mean: 80.0, 62.7, and 32.6 ng/mL for CP class A, B, and C, respectively) were inversely correlated with the CP score, and the L-IGF1 group had lower 2 years survival rates than the H-IGF1 group (13). Another study of patients with decompensated cirrhosis (without HCC) revealed that serum IGF-1 levels (median: 70.1, 40.5, and 32.4 ng/mL for CP class A, B, and C, respectively) and IGF-CP scores were associated with 1 year mortality (5). This IGF-CP scoring system more accurately predicted 1 year mortality than the CP score alone. Another study of patients with CP class A and advanced HCC receiving antiangiogenic therapy revealed that low serum IGF-1 levels (<63.6 ng/mL) reduced the progression-free and overall survival rates (17). Our study revealed that the median IGF-1 levels were 62, 43, and 34 ng/mL for CP class A, B, and C, respectively, and 61 and 46 ng/mL for compensated and decompensated cirrhosis, respectively. The IGF-1 predictive value of poor prognosis in patients with cirrhosis (without HCC) may be approximately 40 ng/mL, considering that the cutoff IGF-1 values for the L-IGF1 groups were 41, 47, and 32 ng/mL in all patients and those with compensated and decompensated cirrhosis, respectively.

The MELD and CP scores provide prognostic information for patients with decompensated cirrhosis (18). The MELD score predicts short-term mortality in end-stage liver disease and is useful for determining organ allocation to liver transplantation candidates on transplant waiting lists (19, 20). Meanwhile, the CP score predicts differences in prognosis between patients with CP classes A and B/C (3). However, these scoring systems can hardly identify patients with poor prognoses when limited to those with compensated cirrhosis (6, 18). Therefore, circulating IGF-1 levels may be a useful predictor of long-term prognosis in the compensated phase.

The present study revealed that serum IGF-1 levels were significantly correlated with liver functional reserve-related factors, which was consistent with previous studies (10, 13, 21, 22). Serum IGF-1 levels correlated positively with albumin and PT levels and negatively with total bilirubin level, CP score, and MELD score. Reportedly, serum IGF-1 levels are substantially low in a large percentage of patients with compensated cirrhosis despite normal albumin and prealbumin levels; hence, they may be a more sensitive and earlier indicator of impaired liver function than other conventional parameters (13). As described above, patients with compensated cirrhosis must inhibit the progression to decompensation. The MELD and CP scores are widely used to estimate liver functional reserve; however, these provide little information on the risk of developing decompensation (18). The present study identified serum IGF-1 levels as the only significant factor associated with decompensation development. The L-IGF1_com_ group had significantly higher cumulative incidence rates of decompensation than the H-IGF1_com_ and I-IGF1_com_ groups. Intriguingly, the H-IGF1_com_ group demonstrated no decompensation. Therefore, serum IGF-1 levels may be a simple and useful indicator of liver functional reserve and the risk of developing decompensation.

Hepatic stellate cells (HSCs), which are activated by oxidative stress, proinflammatory cytokines, and autophagy, participate in liver regeneration and fibrosis (23). Meanwhile, cellular senescence of activated HSCs suppresses liver fibrogenesis (24). IGF-1 administration in mouse models with methionine-choline-deficient diet-induced nonalcoholic steatohepatitis and dimethylnitrosamine-induced cirrhosis ameliorated hepatic steatosis, inflammation, and fibrosis (25). Additionally, IGF-1 induced HSCs into cellular senescence *in vitro and in vivo* and inhibited fibrogenesis in a p53-dependent manner. Similarly, IGF-1 administration improved liver function (increased albumin, total protein, and coagulation factor levels) and reduced oxidative liver damage and fibrosis in rat models with CCl_4_-induced cirrhosis (26). Preoperative serum IGF-1 and IGF-binding protein-3 levels in the cirrhosis group who underwent liver transplantation were lower than those in the control group, but their postoperative levels recovered to normal (27). A pilot study of patients with cirrhosis revealed that IGF-1 administration for 4 months increased serum albumin levels and improved energy metabolism, as assessed by resting energy expenditure (28). These basic and clinical research findings indicate that IGF-1 which is largely produced in hepatocytes is closely involved in hepatic inflammation and fibrosis regulation; hence, advanced disease stage and impaired liver functional reserve reduce IGF-1 levels, which may in turn exacerbate the disease conditions and cause poor prognosis.

This study has some limitations. First, this was a retrospective, small-scale study; therefore, prospective, large-scale studies are needed to confirm our findings. Second, we were unable to assess the IGF-binding protein levels, which regulate the biological activity of IGF-1 and may be associated with liver functional reserve (8, 9, 21). Finally, serum IGF-1 levels were measured only at baseline, whereas longitudinal changes in serum IGF-1 levels were not investigated; thus, the differences in the longitudinal changes between patients with and without decompensation development or between patients with good and poor prognoses remain unclear.

## 5. Conclusion

In conclusion, low serum IGF-1 levels were associated with decompensation development and poor long-term prognosis in patients with compensated cirrhosis. Therefore, IGF-1 may be useful for predicting decompensation-related events and should be regularly monitored in the management of compensated cirrhosis.

## Data availability statement

The original contributions presented in the study are included in the article/Supplementary material, further inquiries can be directed to the corresponding authors.

## Ethics statement

The studies involving human participants were reviewed and approved by The ethics committees of the Jikei University School of Medicine (approval number: 34-021) and Fuji City General Hospital (approval number: 279). Written informed consent for participation was not required for this study in accordance with the national legislation and the institutional requirements.

## Author contributions

CS participated in the conception and design of the study. CS, TK, KU, MN, TO, and YT acquired, analyzed, and interpreted the data. CS and AT drafted the manuscript. MS and AT interpreted the data and revised the manuscript. AT substantively revised and completed the manuscript. All authors contributed to the article and approved the submitted version.

## Conflict of interest

The authors declare that the research was conducted in the absence of any commercial or financial relationships that could be construed as a potential conflict of interest.

## Publisher’s note

All claims expressed in this article are solely those of the authors and do not necessarily represent those of their affiliated organizations, or those of the publisher, the editors and the reviewers. Any product that may be evaluated in this article, or claim that may be made by its manufacturer, is not guaranteed or endorsed by the publisher.
