# Supplementary material for: Insulin-like growth factor 1 predicts decompensation and long-term prognosis in patients with compensated cirrhosis
Source: Front Med (Lausanne). 2023 Jul 24;10:1233928. doi: 10.3389/fmed.2023.1233928 (PMC10405075; doi:10.3389/fmed.2023.1233928)
Supplement: Supplementary file 1 [file Table_1.DOCX]

**Table S1. Univariate analysis of factors associated with mortality in all patients**

| Variable | HR (95% CI) | *p*-value |
| --- | --- | --- |
| Gender (Man) | 1.391 (0.637–3.040) | 0.407 |
| Age (years) | 1.002 (0.972–1.034) | 0.881 |
| BMI (kg/m^2^) | 0.931 (0.845–1.025) | 0.143 |
| Etiology | 1.140 (0.770–1.688) | 0.512 |
| Decompensated cirrhosis | 6.664 (2.955–15.029) | < 0.001 |
| Child-Pugh score | 1.695 (1.454–1.977) | < 0.001 |
| MELD score | 1.202 (1.106–1.305) | < 0.001 |
| Total bilirubin (mg/dL) | 1.606 (1.296–1.991) | < 0.001 |
| Albumin (g/dL) | 0.376 (0.274–0.515) | < 0.001 |
| Creatinine (mg/dL) | 1.221 (0.586–2.543) | 0.595 |
| Sodium (mEq/L) | 0.796 (0.688–0.920) | 0.002 |
| Prothrombin time (%) | 0.969 (0.951–0.987) | < 0.001 |
| IGF-1 (ng/mL) | 0.952 (0.932–0.973) | < 0.001 |
| M2BPGi (C.O.I.) | 1.208 (1.125–1.297) | < 0.001 |

BMI, body mass index; CI, confidence interval; C.O.I., cut-off index; HR, hazard ratio; IGF-1, insulin-like growth factor 1; M2BPGi, Mac-2 binding protein glycosylation isomer; MELD, model for end-stage liver disease.

**Table S2. Univariate analysis of factors associated with mortality in patients with compensated cirrhosis**

| Variable | HR (95% CI) | *p*-value |
| --- | --- | --- |
| Gender (Man) | 1.812 (0.365–8.989) | 0.467 |
| Age (years) | 0.971 (0.916–1.029) | 0.316 |
| BMI (kg/m^2^) | 0.944 (0.795–1.121) | 0.509 |
| Etiology | 1.287 (0.602–2.750) | 0.515 |
| Child-Pugh score | 1.880 (0.722–4.898) | 0.196 |
| MELD score | 1.055 (0.800–1.393) | 0.704 |
| Total bilirubin (mg/dL) | 2.381 (0.480–11.800) | 0.288 |
| Albumin (g/dL) | 0.730 (0.287–1.854) | 0.508 |
| Creatinine (mg/dL) | 0.093 (0.004–2.367) | 0.150 |
| Sodium (mEq/L) | 0.934 (0.657–1.293) | 0.680 |
| Prothrombin time (%) | 0.957 (0.915–1.001) | 0.054 |
| IGF-1 (ng/mL) | 0.927 (0.884–0.972) | 0.002 |
| M2BPGi (C.O.I.) | 1.418 (1.008–1.996) | 0.045 |

BMI, body mass index; CI, confidence interval; C.O.I., cut-off index; HR, hazard ratio; IGF-1, insulin-like growth factor 1; M2BPGi, Mac-2 binding protein glycosylation isomer; MELD, model for end-stage liver disease.

**Table S3. Univariate analysis of factors associated with mortality in patients with decompensated cirrhosis**

| Variable | HR (95% CI) | *p*-value |
| --- | --- | --- |
| Gender (Man) | 1.343 (0.547–3.298) | 0.520 |
| Age (years) | 1.031 (0.991–1.037) | 0.135 |
| BMI (kg/m^2^) | 0.990 (0.872–1.124) | 0.878 |
| Etiology | 1.114 (0.690–1.800) | 0.658 |
| Child-Pugh score | 1.548 (1.218–1.968) | < 0.001 |
| MELD score | 1.113 (1.005–1.233) | 0.039 |
| Total bilirubin (mg/dL) | 1.286 (1.002–1.651) | 0.048 |
| Albumin (g/dL) | 0.104 (0.041–0.265) | < 0.001 |
| Creatinine (mg/dL) | 1.233 (0.638–2.383) | 0.534 |
| Sodium (mEq/L) | 0.878 (0.732–1.054) | 0.162 |
| Prothrombin time (%) | 0.997 (0.976–1.020) | 0.822 |
| IGF-1 (ng/mL) | 0.974 (0.951–0.998) | 0.034 |
| M2BPGi (C.O.I.) | 1.086 (0.985–1.196) | 0.097 |

BMI, body mass index; CI, confidence interval; C.O.I., cut-off index; HR, hazard ratio; IGF-1, insulin-like growth factor 1; M2BPGi, Mac-2 binding protein glycosylation isomer; MELD, model for end-stage liver disease.

**Table S4. Univariate analysis of factors associated with decompensation development**

| Variable | HR (95% CI) | *p*-value |
| --- | --- | --- |
| Gender (Man) | 1.432 (0.555–3.692) | 0.458 |
| Age (years) | 0.977 (0.944–1.013) | 0.205 |
| BMI (kg/m^2^) | 0.972 (0.875–1.079) | 0.594 |
| Etiology | 1.303 (0.823–2.064) | 0.259 |
| Child-Pugh score | 2.932 (1.623–5.295) | < 0.001 |
| MELD score | 1.110 (0.955–1.290) | 0.174 |
| Total bilirubin (mg/dL) | 3.065 (1.178–7.975) | 0.022 |
| Albumin (g/dL) | 0.603 (0.373–0.974) | 0.039 |
| Creatinine (mg/dL) | 0.744 (0.211–2.624) | 0.646 |
| Sodium (mEq/L) | 0.943 (0.775–1.147) | 0.555 |
| Prothrombin time (%) | 0.958 (0.932–0.985) | 0.003 |
| IGF-1 (ng/mL) | 0.942 (0.917–0.968) | < 0.001 |
| M2BPGi (C.O.I.) | 1.348 (1.097–1.655) | 0.004 |

BMI, body mass index; CI, confidence interval; C.O.I., cut-off index; HR, hazard ratio; IGF-1, insulin-like growth factor 1; M2BPGi, Mac-2 binding protein glycosylation isomer; MELD, model for end-stage liver disease.
