# Supplementary figures and images for: Insulin-like growth factor 1 predicts decompensation and long-term prognosis in patients with compensated cirrhosis
Source: Front Med (Lausanne). 2023 Jul 24;10:1233928. doi: 10.3389/fmed.2023.1233928 (PMC10405075; doi:10.3389/fmed.2023.1233928)

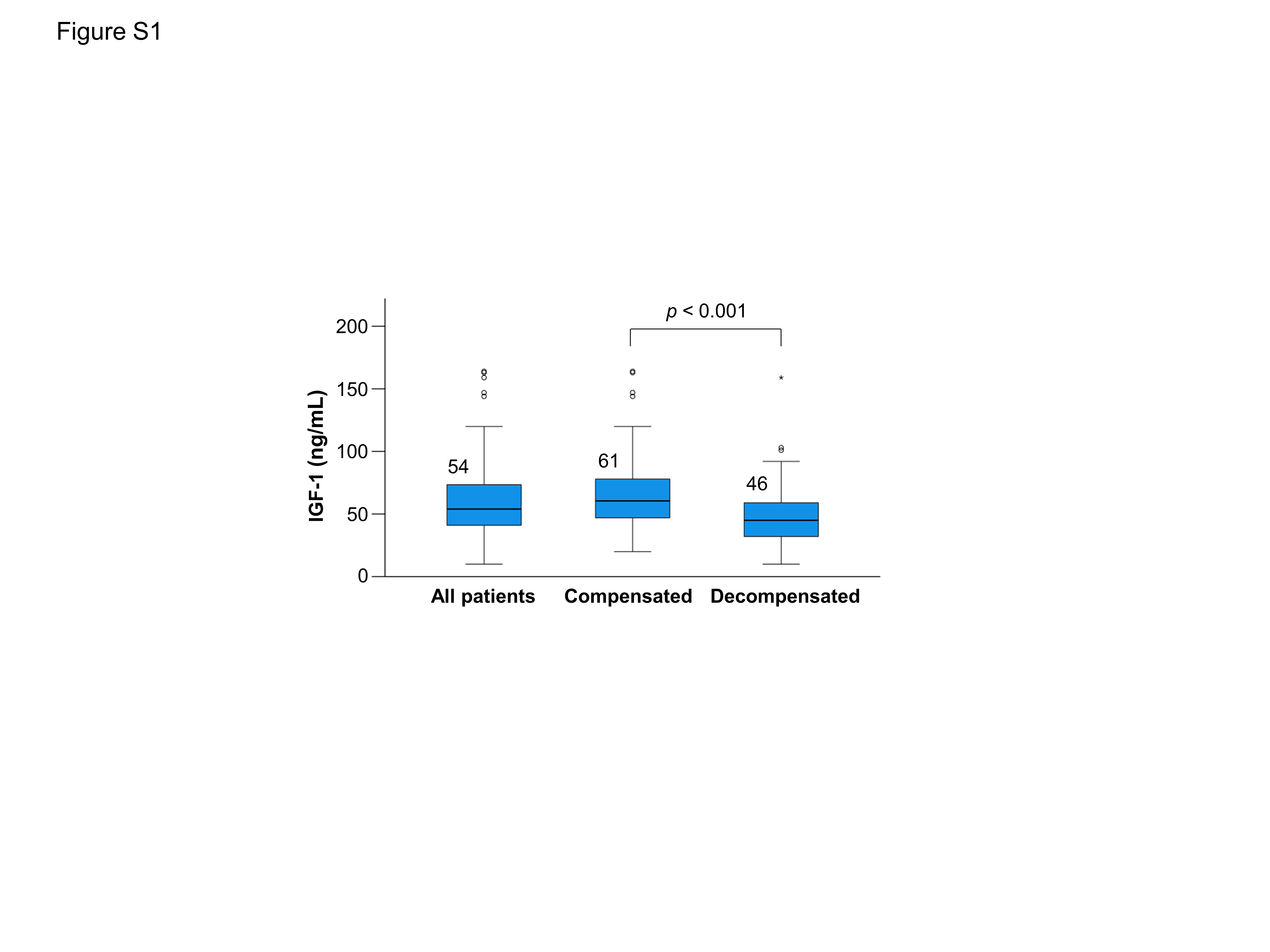

Supplement: Supplementary file 2 [file Image_1.tif]

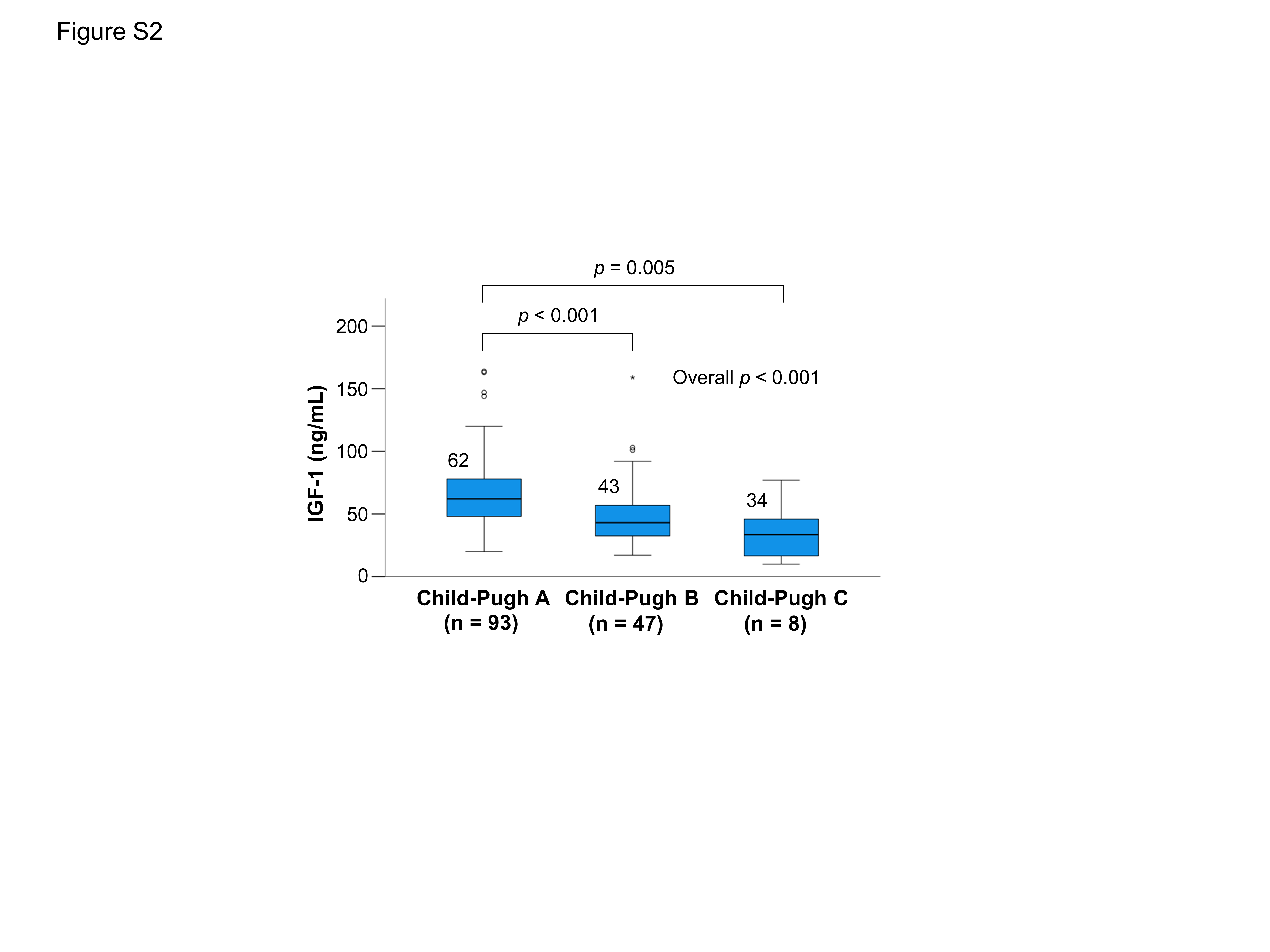

Supplement: Supplementary file 3 [file Image_2.TIF]

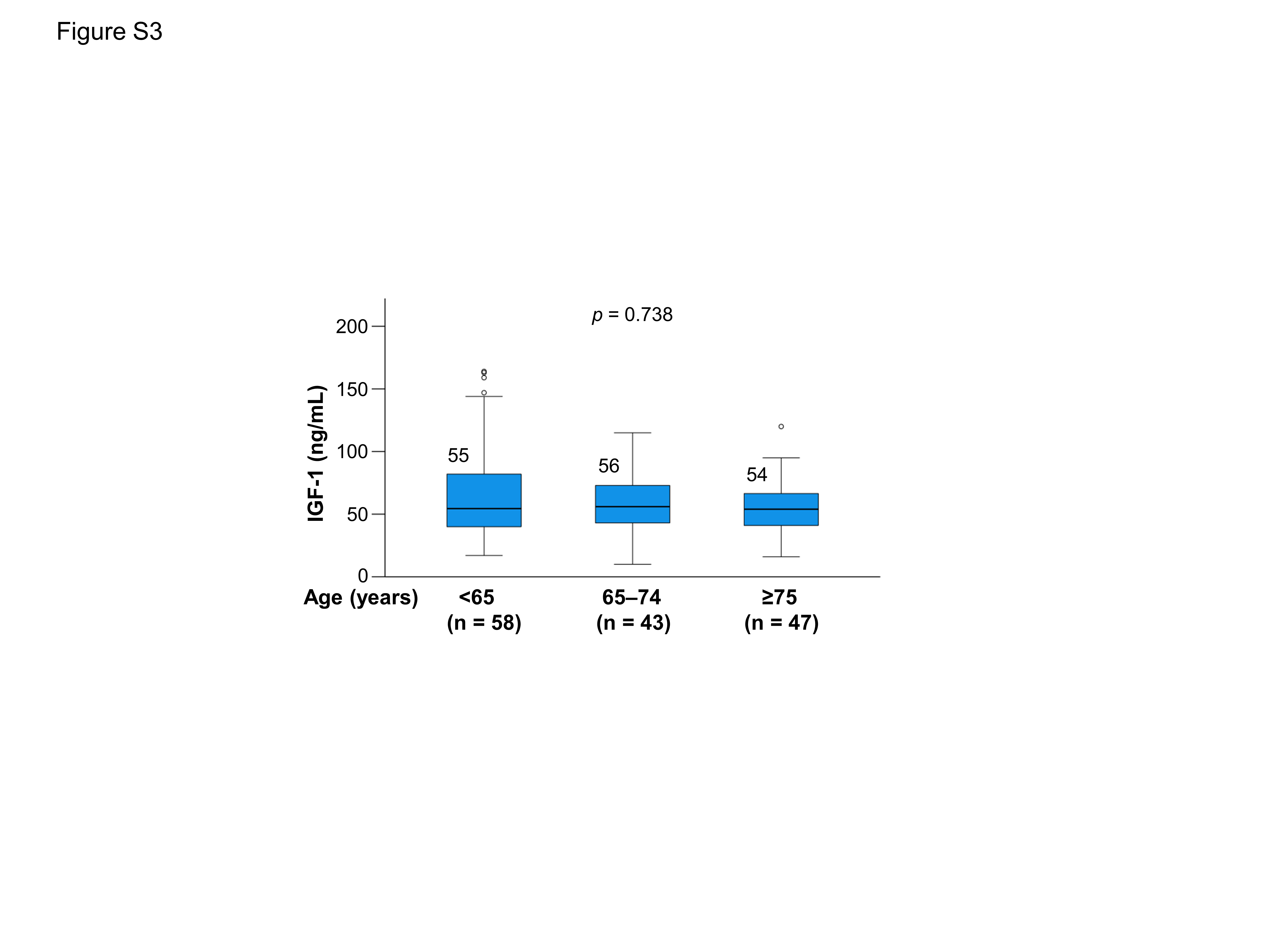

Supplement: Supplementary file 4 [file Image_3.TIF]

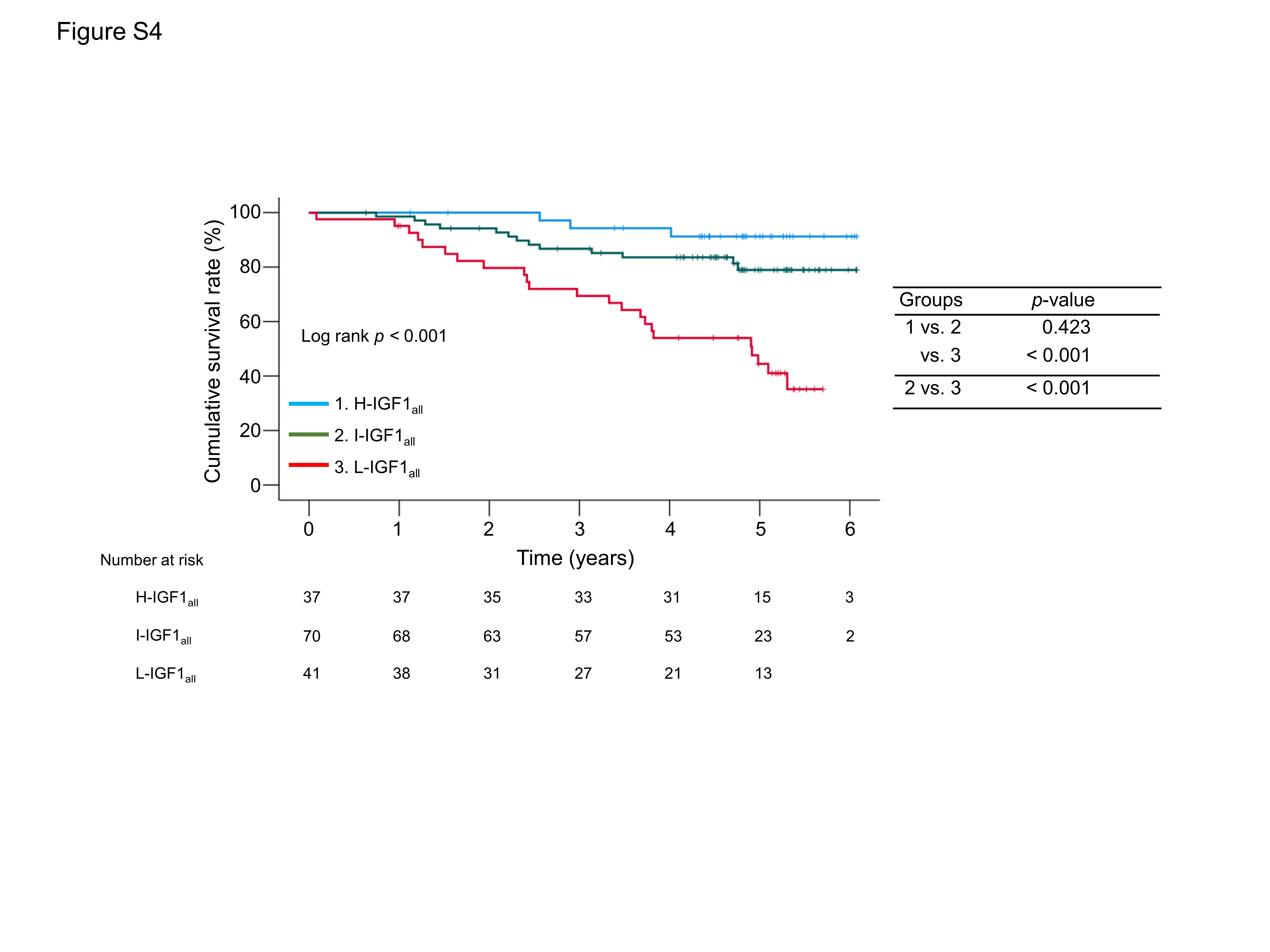

Supplement: Supplementary file 5 [file Image_4.tif]

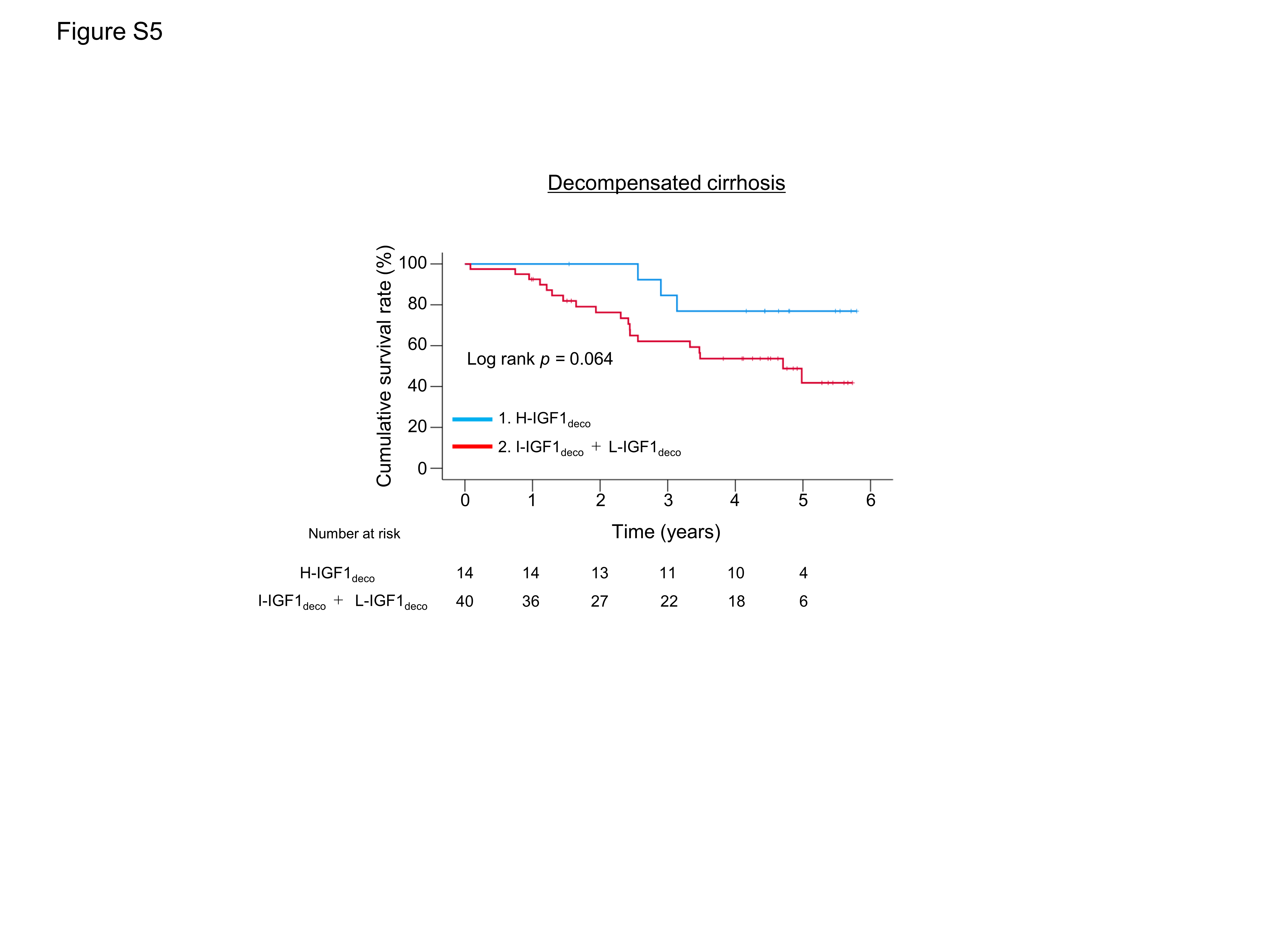

Supplement: Supplementary file 6 [file Image_5.tif]
